# Prevalence and magnitude weight loss among collegiate wrestlers: have practices changed?

**DOI:** 10.1080/15502783.2025.2550144

**Published:** 2025-08-28

**Authors:** Andrew R. Jagim, Ward C. Dobbs, Craig A. Horswill, Eric Twohey, Jennifer B. Fields, Margaret T. Jones

**Affiliations:** a Mayo Clinic Health System, Sports Medicine, La Crosse, WI, USA; b University of Wisconsin – La Crosse, Department of Exercise and Sport Science, La Crosse, WI, USA; c University of Wisconsin – La Crosse, Department of Physical Therapy, La Crosse, WI, USA; d University of Illinois Chicago, Emertius, Department of Kinesiology and Nutrition, Chicago, IL, USA; e Mayo Clinic, Physical Medicine and Rehabilitation, Rochester, MN, USA; f University of Connecticut, Department of Nutritional Sciences, Storrs, CT, USA; g George Mason University, Sport, Recreation and Tourism Management, Fairfax, VA, USA

**Keywords:** Weight class, body fat percentage, competition, wrestling

## Abstract

**Background:**

The purpose of this study was to examine the percentage of wrestlers who compete in their minimal weight class (MWC), and the magnitude of weight loss.

**Methods:**

Data from the 2023–2024 collegiate season were retrospectively analyzed resulting in a sample of 9,638 collegiate men wrestlers from the National Association of Intercollegiate Athletics (NAIA, *n* = 1,904) and all three divisions of the National Collegiate Athletics Association (NCAA, *n* = 7,734). All wrestlers completed skinfold assessments for weight certification at the start of the competition season. A body fat percent threshold of 5% was used to determine minimal wrestling weight (MWW). The lowest recorded weight class (LRW) achieved by each wrestler during the season was also recorded and used to determine magnitude of weight loss.

**Results:**

Out of the 4605 (53.2%) of wrestlers who competed in their MWC, their average amount of weight loss was significantly higher than those who did not compete in their MWC (8.7 ± 5.6 vs. 7.1 ± 6.5 lbs.; *p* < 0.001), yet they had an initial lower BF% (12.7 ± 2.9 vs. 17.5 ± 4.8 %; *p* < 0.001). On average, wrestlers weighed 17.8 ± 9.9 (95% CI: 17.6, 18.0; effect size [ES] = 1.8) lbs. more at the time of weight certification compared to their MWW. On average, wrestlers lost 8.0 ± 6.5 (95% CI: 7.9, 8.1; ES = 1.2) lbs. between the span from weight certification and LRW during the season. This represented an average weight loss equal to 4.6 ± 3.6% of their initial body weight. Throughout the season, wrestlers competed at a weight class that was, on average (mean ± SD), 9.8 ± 8.2 lbs. higher than their MWW (95% CI: 9.6, 9.9 lbs. *p* < 0.001; ES = 1.2).

**Conclusions:**

A total of 90% of BF% values were above 10% body fat indicating that the vast majority would qualify for weight reduction to achieve MWW at 5% BF. However, only slightly more than half of the wrestlers competed in their MWC. This required a greater magnitude of weight loss while starting from a leaner state compared to those who did not compete in their MWC. Notable differences in body fat percentage were observed between weight class divisions, with heavier weight classes exhibiting higher body fat percentages.

